# Using craniofacial characteristics to predict optimum airway pressure in obstructive sleep apnea treatment^☆^

**DOI:** 10.1016/j.bjorl.2018.10.012

**Published:** 2018-12-12

**Authors:** Thays Crosara Abrahão Cunha, Thais Moura Guimarães, Fernanda R. Almeida, Fernanda L.M. Haddad, Luciana B.M. Godoy, Thulio M. Cunha, Luciana O. Silva, Sergio Tufik, Lia Bittencourt

**Affiliations:** a Universidade Federal de São Paulo (Unifesp), Departamento de Psicobiologia, São Paulo, SP, Brazil; b University of British Columbia, Faculty of Dentistry, Vancouver, Canada; c Universidade Federal de Uberlândia (UFU), Laboratório de Função Pulmonar, Departamento de Pneumologia, Uberlândia, MG, Brazil

**Keywords:** Continuous positive airway pressure, Obstructive sleep apnea, Snoring, Cephalometry, Pressão positiva contínua nas vias aéreas, Apneia obstrutiva do sono, Ronco, Cefalometria

## Abstract

**Introduction:**

Manual titration is the gold standard to determinate optimal continuous positive airway pressure, and the prediction of the optimal pressure is important to avoid delays in prescribing a continuous positive airway pressure treatment.

**Objective:**

To verify whether anthropometric, polysomnographic, cephalometric, and upper airway clinical assessments can predict the optimal continuous positive airway pressure setting for obstructive sleep apnea patients.

**Methods:**

Fifty men between 25 and 65 years, with body mass indexes of less than or equal to 35 kg/m^2^ were selected. All patients had baseline polysomnography followed by cephalometric and otolaryngological clinical assessments. On a second night, titration polysomnography was carried out to establish the optimal pressure.

**Results:**

The average age of the patients was 43 ± 12.3 years, with a mean body mass index of 27.1 ± 3.4 kg/m^2^ and an apnea–hypopnea index of 17.8 ± 10.5 events per hour. Smaller mandibular length (*p* = 0.03), smaller atlas–jaw distance (*p* = 0.03), and the presence of a Mallampati III and IV (*p* = 0.02) were predictors for higher continuous positive airway pressure. The formula for the optimal continuous positive airway pressure was: 17.244 − (0.133 × jaw length) + (0.969 × Mallampati III and IV classification) − (0.926 × atlas–jaw distance).

**Conclusion:**

In a sample of male patients with mild-to-moderate obstructive sleep apnea, the optimal continuous positive airway pressure was predicted using the mandibular length, atlas–jaw distance and Mallampati classification.

## Resumo

**Introdução:**

A titulação manual é o padrão-ouro para determinar a pressão ideal para o tratamento com a pressão positiva contínua nas vias aéreas; e a predição da pressão ideal é importante para evitar retardos na sua prescrição.

**Objetivo:**

Verificar se as avaliações clínicas antropométricas, polissonográficas, cefalométricas e das vias aéreas superiores podem predizer a configuração ideal da pressão do aparelho de pressão positiva contínua nas vias aéreas para pacientes com apneia obstrutiva do sono.

**Método:**

Foram selecionados 50 homens entre 25 e 65 anos, com índice de massa corporal menor ou igual a 35 kg/m^2^. Todos os pacientes fizeram polissonografia basal, seguida de avaliações clínicas cefalométricas e otorrinolaringológicas. Na segunda noite, foi feita polissonografia de titulação para estabelecer a pressão ideal.

**Resultados:**

A média de idade dos pacientes foi de 43 ± 12,3 anos, com índice de massa corporal médio de 27,1 ± 3,4 kg/m^2^ e índice de apneia-hipopneia de 17,8 ± 10,5 eventos por hora. Menor comprimento mandibular (*p* = 0,03), menor distância atlas-maxila (*p* = 0,03) e a presença de Mallampati III e IV (*p* = 0,02) foram preditores de pressão mais elevada. A fórmula para a pressão positiva contínua nas vias aéreas foi: 17,24 − (0,133 × comprimento da mandíbula) + (0,969 × classificação de Mallampati III e IV) − (0,926 × distância atlas-mandíbula).

**Conclusão:**

Em uma amostra de homens com apneia obstrutiva do sono leve a moderada, a pressão positiva contínua nas vias aéreas foi predita com o comprimento mandibular, a distância atlas-mandíbula e a classificação de Mallampati.

## Introduction

Continuous positive airway pressure (CPAP) is the most common and effective treatment for patients with obstructive sleep apnea.^1^ Manual titration in a sleep laboratory during full night polysomnography (PSG) is the gold standard to determinate optimal CPAP pressure.^2^ However, the high cost of PSG and difficult accessibility to sleep laboratories may limit the treatment. Therefore, other alternatives have been suggested, such as split-night studies,^3^ auto-CPAP titration,^4^ home-based sleep diagnostic studies^5^, ^6^ and predictive equations to determine CPAP pressure.^7^, ^8^, ^9^

Predictive equations to establish CPAP pressure include factors such as body mass index (BMI), neck circumference, apnea–hypopnea index (AHI), oxyhemoglobin saturation, age and gender.^8^, ^10^, ^11^, ^12^, ^13^, ^14^, ^15^ To the best of our knowledge, only two studies have used cephalometric characteristics to establish the optimal CPAP pressure.^16^, ^17^

Thus, the objective of the present study is to verify whether anthropometric, polysomnographic, cephalometric, and upper airway clinical assessments can predict the optimal CPAP pressure setting for OSA patients.

## Materials and methods

### Sample

The sample was selected from the out clinic patients of the Associação Fundo de Incentivo á Pesquisa, São Paulo (Brazil). Male patients between 25 and 65 years, with a BMI less/or equal to 35 kg/m^2^ and an AHI greater than 5 events/h were selected. We postulated that hormonal, anatomical differences and variations in the mechanisms of ventilatory control could influence the pharyngeal collapse process and consequently the requirements for titration of CPAP pressure, justifying the exclusive selection of men in this study. The patients voluntarily signed an informed consent form, and the study protocol was approved by the UNIFESP's research ethics committee (REC 0352/09). All procedures were in accordance with the 1964 Helsinki declaration.

Patients underwent full-night baseline polysomnography (Embla Systems, Inc., Broomfield, CO, USA), lateral cephalography and otolaryngological clinical evaluation. All patients also underwent a second full-night polysomnography for manual titration of CPAP pressure following Kushida's guidelines,^2^ using conventional nasal masks.

### Cephalometric assessment

The image capture was done with patients keeping their teeth in normal occlusion, not swallowing and in natural head posture.^18^ A number of different parameters were evaluated by lateral cephalometry. The used cephalometric variables, as well as their definitions are described in Table 1.

**Table 1:** Summary of variables used for cephalometric analysis – describing the cephalometric points used, their description, as well as the mean and standard deviation of the values found.

Variables	Definition	Mean ± SD
H–MP (mm)	Hyoid – mandibular plane	21.9 ± 4.7
C3–H (mm)	Third vertebrae – hyoid	77.2 ± 8.7
H–RGN (mm)	Hyoid – retrognathia	39.4 ± 4.5
PNS–P (mm)	Soft palate length	38.3 ± 3.2
SPW–SPW (mm)	Soft palate width	9.9 ± 1.8
Space located between the most prominent point of the external wall of the soft palate and the nasopharyngeal posterior wall (mm)	Middle posterior palatal space	12.0 ± 3.5
Vasa–Vasp (mm)	Superior pharyngeal space	15.1 ± 3.7
Space located between the intersection of the occlusal plane and the anterior and posterior nasopharyngeal wall (mm)	Middle pharyngeal space	9.7 ± 3.3
BGo – Goc (mm)	PAS (posterior airway space)	13.3 ± 3.8
C3′–H (mm)	LAS (lower airway space)	12.1 ± 4.8
Atlas – PNS (mm)	Atlas–jaw distance	50.0 ± 3.3
SNA (°)	Position of the mandible in relation to the cranial base	82.5 ± 3.9
SNB (°)	Position of the maxilla in relation to the cranial base	78.6 ± 4.1
ANB (°)	Maxillary/mandibular antero-posterior discrepancy	3.6 ± 2.6
Go-Me (°)	Mandibular length	73.5 ± 3.7
FMA (°)	Shows the relationship between mandibular and Frankfurt planes	23.8 ± 7.0

### Otolaryngological assessment

The clinical upper airway assessment was performed by a trained otorhinolaryngologist according to the method of Zonato et al. (2003).^19^ Two otorhinolaryngologists with certification in Sleep Medicine were responsible for this evaluation.

We considered nasal conditions unfavorable, when one of these parameters were present: septum deviation Grade II or III; turbinate hypertrophy associated with rhinitis or complaint of obstruction of nose; septum deviation Grade I associated with rhinitis or complaint of obstruction of nose.

Oropharynx was considered unfavorable when one of the following three parameters existed: web palate; more posterior or thick palate; long or thick uvula; medial pillars; tonsils Grade III or IV.

Mallampati was considered unfavorable when modified Mallampati Class III or IV were present.

### Polysomnography

Full-night polysomnography was carried out for the baseline assessment, and the full-night polysomnography for manual titration of CPAP was performed according to the guidelines described by Kushida et al. (2008).^2^ The CPAP device used was REMstar Plus, with a nasal mask and the polygraph Alice 6.

### Statistical analysis

For the elaboration of the equation, the following statistical methodology was adopted: the correlation coefficient was calculated using Pearson's correlation for all independent variables and for the ideal CPAP pressure. The variables that presented a statistically significant correlation or presented theorical relathionship with CPAP pressure were considered as potential predictors. A multivariate linear regression analysis was performed, using dummies variable that included categorical variable as predictor, after obtaining the predictive formula. The multiple regression procedures estimated the linear equation. To confirm this findings, the *t*-test was performed to compare between the pressure measured by the proposed formula and by manual titration in polysomnography.

SPSS version 18.0 (SPSS Inc., Chicago, IL) software was used, and statistical significance was set at *p* ≤ 0.05.

## Results

Demographic and polysomnographic data are presented in Table 1. From the otolaryngological assessment, 28% of the patients met the criteria for unfavorable craniofacial and pharyngeal structures, 44% met the criteria for unfavorable nasal structures, and 70% exhibited Mallampati Type III or Type IV. The descriptions and mean values of the cephalometric parameters are shown in Table 2.

**Table 2:** Anthropometric and polysomnographic characteristics from 50 OSA patients evaluated in this study.

Assessed parameters	Mean ± SD
Age (years)	43 ± 12
BMI (kg/m^2^)	27.1 ± 3.4
Neck Circumference (cm)	40.7 ± 3.1
AHI (events/h)	17.8 ± 10.5
Baseline SpO_2_ (%)	95.5 ± 1.2
Average SpO_2_ (%)	94.6 ± 1.6
Minimum SpO_2_ (%)	86.1 ± 4.4
OCPAP (cm H_2_O)	8.6 ± 2.1

BMI, body mass index; AHI, apnea–hypopnea index, SpO_2_, oxyhemoglobin saturation; OCPAP, optimal CPAP pressure measured by PSG titration.

**Figure 1:**
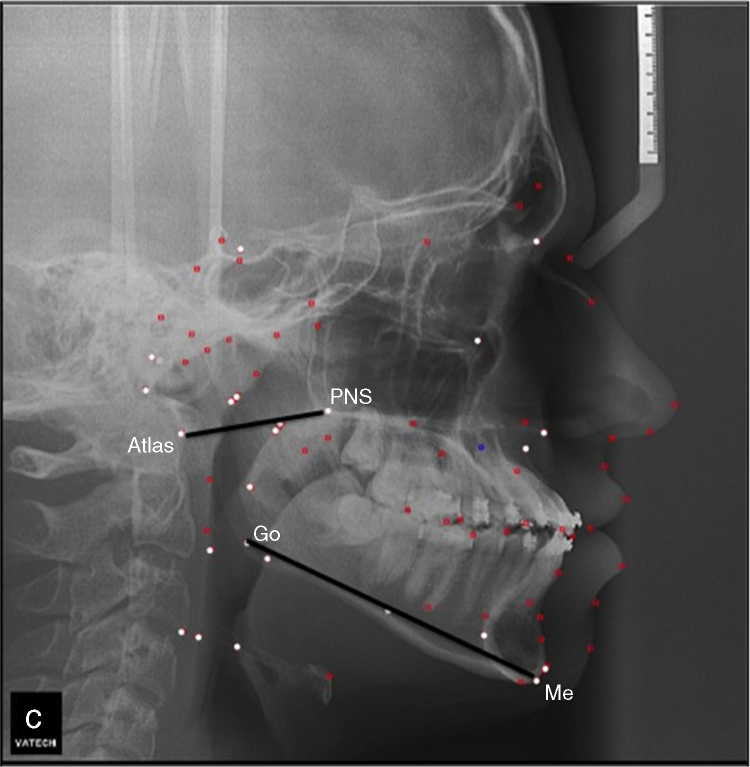
Cephalometric image exemplifying the measures identified as significant.

In regression analyses, lower mandibular length, lower atlas–jaw distance and modified Mallampati III and IV were considered predictors to higher CPAP pressure (Fig. 1), since adjusting for neck circumference, BMI, AHI, age and oxihemoglobin saturations (Table 3).

**Table 3:** Statistical analysis – linear regression to predict CPAP pressure in 50 OSA patients.

Model	*B*	SIG
Mandibular length (mm)	−0.54	0.03^a^
Atlas–jaw distance (mm)	−0.87	0.03^a^
Modified Mallampati II or IV	0.75	0.02^a^
Neck circumference (cm)	−0.14	0.60
BMI (kg/m^2^)	0.03	0.88
*Z* score AHI	0.18	0.60
Basal SpO_2_ (%)	0.78	0.15
Mean SpO_2_ (%)	−0.51	0.23
*Z* score lower SpO_2_ (%)	−0.15	0.70
*Z* score age	−0.23	0.33

Mandibular length, distance between Go–Me point; atlas–jaw distance, distance between At–Enp point; Modified Mallampati, Mallampati III or IV; BMI, body mass index; AHI, apnea–hypopnea index; SpO_2_, oxyhemoglobin saturation.

*R* square explains 75% of the model (*R* squared = 0.756).

a *p* ≤ 0.05, was considered significant.

The following predictor equation best explains the model: optimal CPAP pressure = 17.244 − (0.133 × mandibular length) + (0.969 × modified Mallampati score) − (0.926 × atlas–jaw distance).

**Figure 2:**
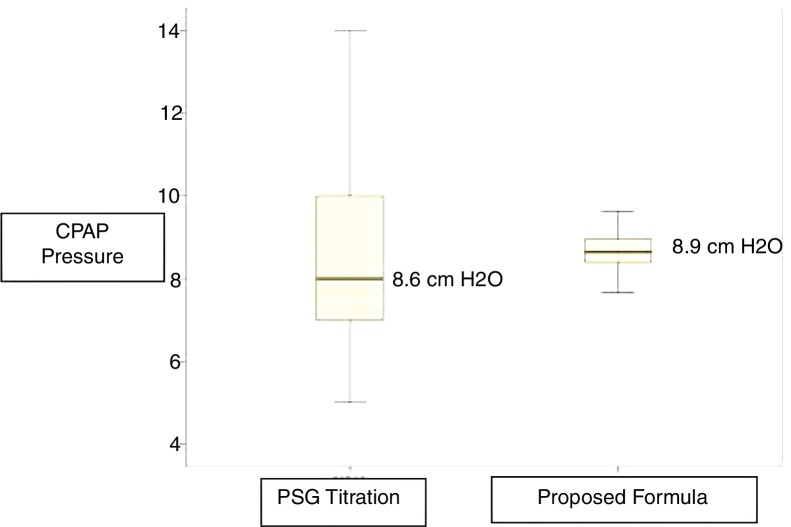
Box-plot derived from the linear regression analyses of the CPAP value using polysomnography and proposed equation, showing statistically similar (Student *t* Test: *p* = 1.00).

The mean of CPAP pressure established by titration was 8.6 cm H_2_O, and the mean of CPAP pressure using the proposed equation was 8.9 cm H_2_O. These values were very close, with the equation yielding a lower variance (Fig. 2).

## Discussion

This is the first study to describe the role of cephalometric variables in the prediction of CPAP pressure in Brazilian individuals. We determined that mandibular length (*p* = 0.03), atlas–jaw distance (*p* = 0.03), and modified Mallampati III and IV classification (*p* = 0.02) were predictors for the CPAP pressure.

The gold standard for determining the ideal CPAP value is a full-night polysomnography using manual titration, in which the pressure setting is progressively adjusted until all obstructive events have been resolved during every sleep stage and for all body positions.^2^

However, the testing period may not be long enough, either due to an insufficient duration of sleep or to the difficulty in achieving the appropriate pressure. Moreover, this method is expensive and demands significant time and intensive labor by trained technicians, contributing to a delayed prescription of this therapy.^20^ Thus, new alternatives have been suggested, such as split-night studies,^4^ auto-CPAP titration,^21^ home-based sleep diagnostic studies,^5^, ^6^ alternatively they can be domiciled with automatic CPAP, respecting the indications and counter indications of the automatic devices,^22^ and more recently, predictor equations for CPAP that are derived from demographic, anthropometric, and polysomnographic parameters.^7^, ^8^, ^9^ Several predictor equations for CPAP pressure have been developed in different Countries and for various populations.^8^, ^11^, ^16^, ^23^, ^24^, ^25^

### Predictor equations for CPAP pressure

Hoffestein and Miljeteig (1993) were one of the first groups to focus on the importance of developing a predictor equation for CPAP, correlating BMI, AHI, and neck circumference for a Caucasian population.^7^ Their formula was later validated by other authors.^24^, ^26^, ^27^, ^28^ Oliver et al. (2000) suggested using the pressure predicted by this equation exclusively to begin the manual titration in laboratory.^29^ Masa et al. (2004) conducted a multicenter study in Spain with 360 patients who required CPAP therapy.^23^ The patients were randomized into 3 groups: manual titration during full-night polysomnography, CPAP auto-titration, and titration using Hoffestein's equation. The authors concluded that titration using this formula is as effective as the manual titration in patients with severe OSA, lowering costs and significantly decreasing waiting lists.^23^ It is important to emphasize that all previous studies have included patients with severe OSA, which differs from our sample, which included only mild to moderate OSA.

According to Basoglu et al. (2011),^21^ race and ethnicity are important factors for determining patients’ physical characteristics, the severity of OSA, and the CPAP pressure level that is necessary to resolve their apnea and hypopnea. These authors conducted a retrospective study in which 250 patients underwent polysomnography with manual titration. Various combinations of anthropometric and polysomnographic variables were tested. The obtained data a provided the development of a predictor equation for the Turkish population that included only the neck circumference and the oxy-hemoglobin desaturation index.^21^ In contrast, our study did not include patients with severe sleep apnea. Similarly to the Turkish population in Masa's study, the Brazilian population is highly mixed and might require the validation of a specific equation for this population.

Schiza et al. (2011)^24^ described a predictor equation for the Greek population that included BMI, AHI, gender, and smoking history. This prospective study compared the manual titration to Hoffestein's equation with an equation developed by the group.^24^ Although both equations predicted the ideal CPAP pressure, Hoffestein's equation was less effective, as determined in other populations.^15^, ^26^, ^27^, ^28^ Lin et al. (2003) formulated a predictor equation based on the BMI and AHI of Taiwanese individuals and determined that neck circumference was not relevant for the Asian population,^8^ a fact that was also observed in our study. Likewise, in a retrospective study, Choi included 202 Koreans, 182 of whom were males with mild, moderate, or severe OSAS; a predictor equation was generated that similarly included only the two variables, BMI and AHI.^11^

### Craniofacial characteristics

Many studies have demonstrated that OSA patients exhibit important craniofacial alterations, which could explain the presence of OSA even in non-obese patients, especially in the Japanese population, where previous studies on cephalometric variables such as lower cranial base flexion^17^ and the position of the hyoid bone in relation with the chin^16^ were correlated with CPAP pressure.

Sforza et al. (1995) reported that the length of the soft palate affected the efficacy of CPAP in 22 French patients with OSA.^30^ However, because the soft palate is a component of the soft tissue, it is possibly influenced by obesity. This finding contrasts with our data in which the equation was not influenced by BMI, but was influenced by Mallampati score. In our study, the required CPAP pressure presented a positive correlation to the Mallampati III and IV classification, suggesting that the smaller volume of the upper airway requires higher CPAP pressure.

It has been suggested that retrognathia and obesity are associated with an inferior position of the hyoid bone.^31^ This fact might explain the negative relationship between the optimum CPAP pressure and mandibular length.

In the present study, we observed that the atlas–jaw distance and mandibular length showed a negative correlation with the optimal CPAP pressure. Even from the linear parameter, we could infer that there is a negative correlation between superior airway size and CPAP pressure.

Akahoshi et al. (2009)^16^ analyzed anthropometric, polysomnographic and cephalometric characteristics as predictors of therapeutic CPAP pressure. In the study, all of the patients had severe OSA and were moderately obese. When the cephalometric variables were combined with the polysomnographic parameters and the BMI, the therapeutic CPAP pressure was predicted more accurately, suggesting that craniofacial characteristics are critical to the pathogenesis of OSA in Asian populations.^16^

It should be noted that predictive equations cannot be a substitute for manual titration, but they could be used as an auxiliary procedure, helping to simplify the titration process and to decrease the number of changes of the pressure settings. However, predictive equations may be used as an alternative to manual titration in situations where a laboratory titration is either not possible (due to immobility, safety concerns, or critical health conditions) or when high costs, prolonged titration times, or lengthy waiting lists prevent or delay titration in sleep laboratories.

The study has some limitation, since the formula needs to be validated on a separate set of patients; we included only male patients and in this protocol, and an additional radiograph exam was required.

## Conclusion

In a sample of non-obese male patients with mild-to-moderate OSAS, optimal CPAP pressure was predicted by mandibular length, atlas–jaw distance and the modified Mallampati classification.

## Funding

This work was supported by *Associação Fundo de Incentivo à Pesquisa* (AFIP); *Fundação de Amparo à Pesquisa do Estado de São Paulo* (FAPESP); and the 10.13039/501100003593*Conselho Nacional de Desenvolvimento Científico e Tecnológico* (CNPq).

## Conflicts of interest

The authors declare no conflicts of interest.
